# Supplementary material for: Effects of Straw Returning and New Fertilizer Substitution on Rice Growth, Yield, and Soil Properties in the Chaohu Lake Region of China
Source: Plants (Basel). 2024 Feb 2;13(3):444. doi: 10.3390/plants13030444 (PMC10857592; doi:10.3390/plants13030444)
Supplement: Supplementary file 1 [file plants-13-00444-s001.zip › plants-2837417-supplementary.pdf]

supplementary material

Effects of Straw Returning and New Fertilizer Substitution on Rice Growth, Yield, and Soil Properties in the Chaohu Lake Region of China

Mei Luo [1], Ying Liu [1], Jing Li [1], Tingfeng Gao [1], Sheng Wu [1], Lei Wu [1], Xijun Lai [2], Hongjun Xu [3], Hongxiang Hu [1,*] and Youhua Ma [1,*]

[1] College of Resources and Environment, Anhui Agricultural University, Hefei 230036, China; meiluo@stu.ahau.edu.cn (M.L.)
[2] Nanjing Institute of Geography and Limnology, Chinese Academy of Sciences, Nanjing 210008, China
[3] Station of Agricultural Environment Protection, Chaohu 238006, China
* Correspondence: huhongxiang@ahau.edu.cn (H.H.); yhma@ahau.edu.cn (Y.M.)

Table S1. Plant height (a), main root length (b), tiller number (c), leaf area index (d), chlorophyll content (e), and aboveground dry weight (f) during observation under five treatments.

Growth stage	*Treatment*	*Plant height (cm)*	*Main root length (cm)*	*Tiller number*	*Leaf area index*	*Chlorophyll content*	*Aboveground dry weight (g plant $^{-1}$)*
	CK	*69.31±3.71*	*15.02±1.36*	*7.00±1.00*	*3.40±0.56*	*32.60±1.51*	*13.95±0.41*
	OF	*73.81±2.48*	*18.20±0.45*	*9.67±1.53*	*4.05±0.24*	*36.27±1.01*	*22.13±4.83*
Mid-tillering stage	*SF*	*76.23±2.92*	*24.96±1.56*	*10.00±2.00*	*3.99±0.43*	*36.42±2.72*	*33.97±2.16*
	SRF	*82.76±9.22*	*20.02±1.19*	*11.00±2.00*	*4.69±0.54*	*38.88±0.39*	*34.49±2.05*
	WSF	*76.03±4.23*	*22.10±0.45*	*10.00±1.00*	*3.91±0.55*	*36.41±0.06*	*30.57±4.48*
Jointing stage	*CK*	*89.97±2.58*	*26.13±3.25*	*10.33±0.58*	*4.00±0.39*	*32.91±0.78*	*25.68±0.66*

	OF	98.42±4.26	28.00±0.69	15.00±2.00	5.04±0.16	35.68±0.60	39.38±2.98
	SF	103.37±1.90	38.4±2.40	15.33±2.08	5.34±0.35	37.5±0.58	49.24±1.52
	SRF	106.20±4.40	30.8±1.83	16.00±2.00	5.45±0.85	37.79±2.88	50.08±1.38
	WSF	104.41±0.87	34.00±0.69	15.33±1.15	5.52±0.58	36.35±0.66	45.81±0.69
Heading stage	CK	112.77±3.21	13.93±3.02	10.33±0.58	4.02±0.39	29.33±1.99	123.88±13.34
	OF	124.28±3.33	15.55±0.61	14.00±1.00	5.06±0.16	32.52±1.25	199.07±22.56
	SF	125.23±0.79	17.97±1.06	14.67±0.58	5.36±0.35	33.59±1.84	202.20±16.34
	SRF	127.95±1.49	20.93±1.55	15.00±1.00	5.47±0.85	33.97±1.33	216.36±10.16
	WSF	125.9±3.69	30.21±1.47	14.33±1.53	5.33±0.07	33.72±1.08	204.48±10.50
Maturity stage	CK	120.4±2.23	13.44±1.65	9.67±1.53	4.06±0.64	14.51±0.73	121.74±8.56
	OF	127.27±	14.85±	13.67±	5.95±	23.06±	184.69±

	1.36	0.40	0.58	0.24	1.36	8.71
SF	128.93±1.47	15.83±1.35	14.00±1.00	6.38±0.51	23.75±1.61	194.89±11.40
SRF	131.33±1.89	15.41±3.48	14.33±1.53	6.52±0.37	24.13±0.48	195.02±5.88
WSF	128.20±1.40	15.18±0.63	14.00±1.00	5.95±0.23	23.64±0.50	193.81±16.66

Note: Data (mean ± standard errors, $n = 3$) with different letters indicate a statistically significant difference based on LSD ($p < 0.05$). The symbols in the following tables and figures are the same as those in this table.

Table S2. Soil pH value at the depths of 0–30, 30–60, and 60–90 cm in the regreening, tillering, heading, and maturity stages under five treatments.

Growth stage	Treatment	pH		
		0 –30 cm	30 –60 cm	60 –90 cm
Mid-tillering stage	CK	5.90±0.44	6.19±0.15	6.18±0.36
	OF	5.86±0.50	6.12±0.11	6.01±0.01
	SF	5.93±0.12	6.02±0.09	6.06±0.03
	SRF	5.85±0.07	5.92±0.10	5.92±0.13
	WSF	5.83±0.06	5.85±0.03	5.90±0.10

	CK	*6.38±0.38*	*6.20±0.41*	*6.33±0.48*
	OF	*6.32±0.08*	*6.46±0.02*	*6.50±0.09*
Jointing stage	*SF*	*6.36±0.10*	*6.36±0.06*	*6.43±0.04*
	SRF	*6.35±0.13*	*6.40±0.09*	*6.49±0.12*
	WSF	*6.46±0.27*	*6.46±0.23*	*6.54±0.19*
	CK	*7.27±0.10*	*7.37±0.05*	*7.46±0.14*
	OF	*6.92±0.09*	*7.10±0.04*	*7.17±0.02*
Heading stage	*SF*	*6.80±0.02*	*7.06±0.02*	*7.10±0.04*
	SRF	*6.79±0.11*	*6.98±0.14*	*7.10±0.04*
	WSF	*6.92±0.15*	*7.05±0.16*	*7.19±0.09*
	CK	*6.83±0.03*	*6.89±0.08*	*7.01±0.04*
	OF	*7.28±0.02*	*7.40±0.20*	*7.51±0.10*
Maturity stage	*SF*	*7.19±0.05*	*7.27±0.06*	*7.38±0.10*
	SRF	*7.11±0.04*	*7.23±0.03*	*7.34±0.03*
	WSF	*7.09±0.07*	*7.08±0.09*	*7.04±0.13*

Table S3. Soil total nitrogen, alkali-hydrolyzable nitrogen, nitrate nitrogen, and ammonium nitrogen at the depths of 0–30, 30–60, and 60–90 cm during observation under five treatments.

Growth stage	Treatment	Soil total nitrogen (g kg^{-1})			Soil alkali-hydrolyzable nitrogen (mg kg^{-1})			Soil nitrate nitrogen (mg kg^{-1})			Soil ammonium nitrogen (mg kg^{-1})		
		0 – 30 cm	30 – 60 cm	60 – 90 cm	0 – 30 cm	30 – 60 cm	60 – 90 cm	0 – 30 cm	30 – 60 cm	60 – 90 cm	0 – 30 cm	30 – 60 cm	60 – 90 cm
Mid-tillering stage	CK	2.21±0.07	1.74±0.16	1.35±0.48	106.77±4.20	46.11±22.24	42.53±1.99	5.97±1.23	6.01±0.26	4.08±0.26	8.19±0.86	8.29±0.72	4.29±0.72
	OF	2.43±0.32	2.31±0.51	1.9±0.93	114.96±14.11	55.81±11.12	46.11±4.20	8.81±1.50	9.00±0.96	7.76±0.87	10.04±1.93	9.91±1.32	8.99±1.05

	SF	2.50±0.22	2.42±0.49	1.97±0.62	116.59±7.29	57.63±4.11	48.53±5.56	11.23±1.97	10.71±0.42	10.26±0.52	13.34±3.83	12.33±1.87	11.91±1.30
	SRF	3.00±0.10	2.78±0.08	2.02±0.13	151.67±7.58	57.03±11.70	65.52±6.30	10.63±0.69	10.58±1.00	10.06±0.47	11.99±2.09	11.69±1.96	10.49±1.03
	WSF	2.52±0.22	2.44±0.68	2.06±0.08	116.47±8.33	89.79±12.78	69.16±3.64	8.96±1.04	8.84±1.43	4.50±0.88	10.92±2.22	9.92±1.29	9.19±0.81
	CK	1.27±0.09	1.41±0.43	1.09±0.52	91.00±7.28	73.04±6.73	56.13±4.47	6.71±1.94	7.01±1.1	6.54±1.03	8.16±1.69	8.38±1.82	7.02±1.83
Jointing stage	OF	1.54±0.32	1.67±0.26	1.44±0.25	104.35±5.56	81.49±21.67	60.67±4.20	9.71±1.56	9.88±1.46	8.35±1.13	10.88±0.88	10.24±0.42	9.10±0.33
	SF	1.81±0.67	2.09±0.28	1.63±0.34	105.56±7.28	83.39±4.20	68.83±22.2	9.49±0.96	9.29±0.65	8.18±0.99	10.68±1.29	11.06±1.06	9.27±0.70
	SRF	2.00±	2.02±	1.68±	137.11±	102.8±7.28	72.17±	10.79±	10.21±	9.61±	12.27±	10.98±	10.05±

		0.24	0.08	0.54	9.16		15.53	1.93	1.36	1.11	1.77	0.81	0.25
	WSF	2.47±0.34	1.91±0.11	1.45±0.16	105.25±3.68	89.79±4.20	69.16±9.63	9.52±1.15	9.22±1.02	8.68±1.23	10.86±1.81	10.12±0.98	9.37±1.25
	CK	1.71±0.51	1.48±0.42	1.11±0.07	181.47±10.26	147.53±5.22	95.9±18.34	7.87±0.52	7.99±0.44	7.57±0.58	9.07±0.3	9.21±0.31	8.61±0.47
	OF	2.36±0.33	1.61±0.12	1.27±0.04	198.89±33.3	157.5±39.31	113.71±6.62	10.44±0.78	10.65±1.3	10.18±1.48	11.54±0.96	11.27±1.25	10.40±0.58
Heading stage	SF	2.37±0.53	1.68±0.08	1.48±0.71	202.15±20.23	158.46±13.23	115.08±4.94	10.48±0.75	9.97±0.44	9.46±0.55	11.66±0.93	11.29±0.81	10.44±1.14
	SRF	2.67±0.42	1.82±0.13	1.59±0.5	231.62±16.42	161.83±18.17	118.72±13.76	10.88±1.25	10.45±0.97	10.04±0.61	11.28±0.31	10.65±0.46	10.19±0.28
	WSF	2.42±0.54	1.79±0.15	1.4±0.38	205.63±14.53	163.28±26.83	115.67±14.72	10.53±1.08	10.13±0.59	9.7±0.22	10.94±0.43	10.68±0.71	9.96±0.78

	CK	2.01±0.13	1.20±0.20	1.04±0.19	151.94±11.66	103.60±17.33	72.42±8.98	5.74±1.71	5.98±0.88	5.53±1.00	6.26±1.23	6.39±1.20	5.91±1.14
	OF	2.24±0.59	1.99±0.11	1.55±0.23	176.14±34.87	129.02±21.39	79.86±4.84	10.55±0.65	10.64±0.59	9.89±0.57	12.1±1.74	11.62±0.81	10.79±0.92
Maturity stage	SF	3.18±0.67	2.06±0.57	1.56±0.38	194.52±32.07	139.14±23.15	80.47±7.50	10.61±0.65	10.24±0.54	9.89±0.39	11.7±0.57	11.26±0.61	10.39±0.66
	SRF	2.81±0.86	2.15±0.26	1.63±0.41	196.70±14.64	141.74±3.95	82.75±18.07	11.34±1.54	10.66±0.67	10.12±0.71	11.71±1.03	11.1±0.70	10.69±0.72
	WSF	3.01±1.11	2.00±0.33	1.59±0.58	182.07±23.37	133.67±7.01	80.66±3.67	10.71±1.04	9.94±0.60	9.64±0.38	10.96±1.16	10.35±0.60	9.78±0.71

Table S4. Soil organic matter, Soil total phosphorus, Soil available phosphorus, and Soil available potassium at the depths of 0–30, 30–60, and 60–90 cm during observation under five treatments.

Growth stage	*Treatment*	*Soil organic matter (g kg^{-1})*			*Soil total phosphorus (g kg^{-1})*			*Soil available phosphorus (mg kg^{-1})*			*Soil available potassium (mg kg^{-1})*		
		0–30 cm	*30–60 cm*	*60–90 cm*	*0–30 cm*	*30–60 cm*	*60–90 cm*	*0–30 cm*	*30–60 cm*	*60–90 cm*	*0–30 cm*	*30–60 cm*	*60–90 cm*
Mid-tillering stage	*CK*	*16.46±0.96*	*9.80±0.51*	*8.76±0.58*	*0.13±0.02*	*0.12±0.01*	*0.10±0.01*	*12.65±1.20*	*10.59±0.39*	*8.31±0.72*	*101.00±1.00*	*75.67±7.51*	*70.00±10.00*
	OF	*21.58±0.32*	*11.93±0.75*	*10.42±1.19*	*0.18±0.02*	*0.17±0.00*	*0.15±0.01*	*16.35±3.51*	*13.86±2.93*	*8.56±0.41*	*108.33±2.89*	*92.33±6.81*	*90.00±10.00*

	SF	22.83±0.38	12.47±0.75	10.54±0.70	0.22±0.06	0.2±0.00	0.16±0.03	16.65±1.72	15.55±3.21	9.44±2.10	115.67±5.13	100.33±4.51	97.33±4.93
	SRF	24.14±0.33	12.85±0.10	10.84±0.53	0.3±0.04	0.23±0.05	0.17±0.02	18.94±1.05	16.73±2.15	10.5±1.27	117.67±6.81	107.33±9.61	94.67±4.16
	WSF	22.83±0.94	13.5±0.32	10.58±0.62	0.23±0.04	0.19±0.03	0.16±0.02	17.49±2.40	14.84±2.41	10.05±5.58	116.33±5.69	96.67±7.02	93.33±9.45
	CK	17.62±1.63	14.95±1.76	12.15±1.23	0.13±0.00	0.11±0.01	0.10±0.00	10.70±0.34	8.53±0.17	5.47±2.40	106.67±3.06	93.33±15.28	90.00±10.00
Jointing stage	OF	21.54±3.11	19.53±0.77	15.58±1.77	0.21±0.02	0.17±0.01	0.15±0.01	11.94±2.65	8.90±0.99	6.00±1.65	123.33±25.17	111.67±7.64	96.67±5.77
	SF	22.09±0.25	19.95±1.05	17.46±0.93	0.19±0.00	0.18±0.01	0.16±0.04	13.57±1.44	9.06±0.93	6.62±1.94	143.33±11.55	120.00±26.46	100.00±20.00
	SRF	25.00±4.	20.63±0.79	17.62±1.8	0.26±0.04	0.20±0.01	0.18±0.01	12.58±1.3	15.25±3.7	8.08±1.61	126.67±11.	123.33±11.	100.00±10.0

		13		*6*				*2*	*7*		*55*	*55*	*0*
	WSF	*22.08±1.53*	*19.69±0.23*	*16.18±0.92*	*0.20±0.01*	*0.19±0.03*	*0.17±0.01*	*15.16±1.77*	*15.13±0.51*	*9.09±4.19*	*140±26.46*	*120.00±10.00*	*93.33±23.09*
	CK	*14.29±4.36*	*120.00±1.69*	*9.38±0.75*	*0.30±0.02*	*0.19±0.03*	*0.17±0.01*	*31.63±4.47*	*16.54±4.19*	*13.24±0.73*	*113.33±5.77*	*110.00±10.00*	*103.33±5.77*
	OF	*19.93±2.15*	*13.57±2.84*	*10.45±2.74*	*0.34±0.08*	*0.24±0.02*	*0.21±0.06*	*33.52±14.23*	*18.42±3.82*	*17.04±3.20*	*127±6.08*	*121.67±7.64*	*110.00±26.46*
Heading stage	*SF*	*20.02±1.70*	*14.26±4.14*	*11.05±1.17*	*0.39±0.04*	*0.29±0.08*	*0.26±0.02*	*39.02±3.86*	*24.14±8.68*	*22.02±2.23*	*136.67±20.82*	*123.67±5.69*	*113.00±11.27*
	SRF	*23.57±0.77*	*15.44±3.58*	*13.32±3.62*	*0.41±0.04*	*0.30±0.04*	*0.23±0.09*	*39.93±3.28*	*26.46±3.82*	*24.66±6.81*	*134.00±6.00*	*133.33±5.77*	*120.00±17.32*
	WSF	*23.02±1.94*	*15.22±3.36*	*12.05±4.03*	*0.36±0.04*	*0.25±0.06*	*0.22±0.05*	*38.17±6.71*	*26.89±3.39*	*20.47±3.16*	*128.00±7.21*	*125.00±32.79*	*113.33±5.77*

	CK	*22.80±2.98*	*16.55±8.25*	*12.79±2.01*	*0.20±0.03*	*0.15±0.03*	*0.14±0.01*	*33.44±5.00*	*17.45±3.39*	*11.09±2.10*	*123.33±11.55*	*111.00±11.53*	*103.33±5.77*
	OF	*26.22±4.04*	*19.67±2.99*	*15.4±2.22*	*0.23±0.02*	*0.19±0.04*	*0.16±0.01*	*40.11±1.71*	*21.14±18.05*	*16.84±5.75*	*136.67±15.28*	*118.00±24.33*	*110.00±26.46*
Maturity stage	*SF*	*28.55±4.26*	*19.96±3.09*	*16.76±4.31*	*0.24±0.03*	*0.21±0.06*	*0.17±0.01*	*41.77±3.38*	*23.07±1.63*	*19.01±0.89*	*142±10.58*	*123.00±6.08*	*113.33±30.55*
	SRF	*30.10±4.56*	*20.25±2.22*	*16.31±1.66*	*0.27±0.03*	*0.23±0.03*	*0.21±0.01*	*44.94±4.57*	*25.15±1.46*	*24.43±5.07*	*148.33±7.64*	*123.33±15.28*	*118.33±12.58*
	WSF	*27.96±1.64*	*20.74±6.45*	*16.53±3.27*	*0.25±0.03*	*0.20±0.02*	*0.18±0.02*	*41.51±4.18*	*21.44±1.63*	*20.52±7.02*	*140.00±10.00*	*120.00±18.03*	*111.67±6.66*

Table S5. Soil neutral phosphatase (a), Soil sucrase (b), Soil urease (c), and Soil catalase activity (d) at the depths of 0–30 cm during observation under five treatments.

Growth stage	*Treatment*	*Soil neutral phosphatase activity (mg g^{-1} d^{-1})*	*Soil sucrase activity (mg g^{-1} d^{-1})*	*Soil urease activity (mg g^{-1} d^{-1})*	*Soil catalase activity (mL g^{-1} $20min^{-1}$)*
Mid-tillering stage	*CK*	*0.53±0.06*	*6.92±0.13*	*0.09±0.00*	*1.56±0.06*
	OF	*1.09±0.03*	*7.96±0.09*	*0.14±0.02*	*1.76±0.07*
	SF	*1.19±0.03*	*8.73±0.12*	*0.20±0.01*	*1.92±0.03*
	SRF	*1.34±0.03*	*10.59±0.05*	*0.24±0.01*	*2.00±0.02*
	WSF	*1.19±0.01*	*9.12±0.05*	*0.2±0.02*	*1.85±0.10*
Jointing stage	*CK*	*0.68±0.03*	*7.48±0.04*	*0.09±0.02*	*1.75±0.07*
	OF	*1.28±0.03*	*8.40±0.02*	*0.12±0.01*	*2.04±0.06*
	SF	*1.33±0.02*	*9.00±0.04*	*0.12±0.01*	*2.49±0.03*
	SRF	*1.52±0.04*	*11.54±0.11*	*0.14±0.01*	*2.88±0.08*
	WSF	*1.49±0.03*	*10.46±0.09*	*0.13±0.01*	*2.48±0.03*
Heading stage	*CK*	*0.78±0.03*	*8.46±0.07*	*0.09±0.01*	*1.66±0.07*

	OF	1.59±0.04	9.88±0.03	0.11±0.01	1.70±0.03
	SF	1.71±0.01	10.77±0.07	0.12±0.01	1.96±0.04
	SRF	1.83±0.05	12.16±0.08	0.14±0.01	2.31±0.03
	WSF	1.74±0.05	11.78±0.06	0.12±0.01	1.78±0.03
Maturity stage	CK	0.62±0.05	8.76±0.07	0.10±0.01	1.45±0.04
	OF	0.86±0.04	11.97±0.15	0.16±0.02	1.59±0.03
	SF	0.94±0.04	13.19±0.18	0.18±0.01	1.70±0.03
	SRF	0.95±0.03	14.27±0.07	0.19±0.02	1.92±0.02
	WSF	0.80±0.02	12.78±0.03	0.16±0.02	1.67±0.02

Table S6. Treatment fertilizer application rates at five treatments.

Treatments	Treatment description	Types of fertilizer	Fertilizer application rates ($kg\ ha^{-1}$)		
			Basal	Tillering	Panical
CK	No fertilizer	Blank	Blank	Blank	Blank
OF	Optimized fertilizer	Urea	220.49	80.18	100.22
		Superphosphate	481.01	Blank	Blank
		Potassium chloride	94.45	Blank	62.97
SF	Straw returning + chemical fertilizers	Straw returning	4497.75	Blank	Blank
		Urea	200.95	73.07	91.34
		Superphosphate	455.90	Blank	Blank

		Potassium chloride	*15.38*	*Blank*	*10.25*
SRF	*Slow-release fertilizer + chemical fertilizers*	*Slow-release fertilizer*	*590.16*	*Blank*	*Blank*
		Urea	*65.25*	*23.73*	*29.66*
		Superphosphate	*87.46*	*Blank*	*Blank*
		Potassium chloride	*Blank*	*Blank*	*Blank*
WSF	*Water-soluble fertilizer + chemical fertilizers*	*Water-soluble fertilizer*	*Blank*	*299.85*	*299.85*
		Urea	*190.59*	*69.30*	*86.63*
		Superphosphate	*480.26*	*Blank*	*Blank*
		Potassium chloride	*90.16*	*Blank*	*60.11*
